# The effects of fixation stability, corneal density, and epithelial hyperplasia on the efficacy of astigmatism correction by transepithelial photorefractive keratectomy

**DOI:** 10.1186/s40662-025-00437-1

**Published:** 2025-05-28

**Authors:** Junjie Yu, Hao Zhou, Minjie Chen, Zhiqiang Yu, Xingtao Zhou, Yishan Qian

**Affiliations:** Department of Ophthalmology, Eye and ENT Hospital, NHC Key Laboratory of Myopia (Fudan University), Fudan University, 83 Fenyang Rd, Shanghai, 200031 People’s Republic of China

**Keywords:** Transepithelial photorefractive keratectomy, Astigmatism, Fixation stability, Corneal density, Epithelial thickness

## Abstract

**Background:**

Transepithelial photorefractive keratectomy (transPRK) can be safely and predictably performed to correct low-to-high astigmatism. This study explored the effects of fixation stability, corneal density (CD), ocular residual astigmatism (ORA), and the surgically-induced change in the epithelial thickness (ΔET) on the efficacy of astigmatism correction by transPRK.

**Methods:**

Eighty-three consecutive patients who underwent transPRK to correct myopia and myopic astigmatism were divided into two groups according to refractive astigmatism [high refractive astigmatism (RA) group: ≥ 2.0 D, n = 31; low RA group: < 2.0 D, n = 52]. Fixation stability was evaluated by measuring the lateral movement of the pupil center on the eye tracker images. The CD was measured using a Pentacam Scheimpflug imaging system, epithelial thickness mapping was performed using optical coherence tomography, and the ORA was determined using vector analysis. Multiple linear regression analyses were performed to identify factors associated with the correction index (CI) and angle of error (AOE).

**Results:**

At 6 months postoperatively, the RA was higher in the high RA group (− 0.66 ± 0.44 D) than in the low RA group (− 0.29 ± 0.29 D, *P* < 0.001), whereas no significant differences were found in CI or AOE between two groups. Multiple linear regression analyses showed that for the low RA group, preoperative anterior CD of the central 2 mm (CD_0-2A_, β =  − 0.482, *P* = 0.011) and ΔET (β = 0.295, *P* = 0.041), were associated with CI, whereas the vector length of the pupil center shift (PCVL, β =  − 0.404, *P* = 0.005) and ΔET (β =  − 0.293, *P* = 0.036) were associated with AOE. For the high RA group, ΔET (β = 0.519, *P* = 0.038) was associated with CI, whereas static cyclotorsion (β =  − 0.493, *P* = 0.040) was associated with AOE. No significant associations were found between ORA and CI or AOE.

**Conclusions:**

Postoperative changes in epithelial thickness were associated with the efficacy of transPRK in both the low and high RA groups, whereas the pupil center shift and anterior CD were associated with the efficacy of transPRK in the low RA group.

## Background

Refined single-step transepithelial photorefractive keratectomy (transPRK) has shown promising results in correcting different types of refractive errors using solutions such as higher beam repetition rates, limbus detection, pupil centroid shift compensation, and eyeball movement compensation. These features precisely target the laser beams to the cornea and reduce higher-order aberrations (HOAs) [1–3].

TransPRK can be safely and predictably performed to correct low-to-high astigmatism [4, 5]. However, few studies have focused on predictors of astigmatic correction efficacy using transPRK. Human eye movement has six degrees of freedom: X/Y lateral shifts, Z leveling, horizontal/vertical rotations, and cyclotorsion (which includes static cyclotorsion and dynamic cyclotorsion) [6, 7]. Despite significant technical advancements in automatic eye-tracking systems, fixation stability and its effect on refractive outcomes remain an issue in corneal refractive surgey [8, 9]. Moreover, transPRK addresses refractive errors by superimposing a defined epithelial thickness profile onto a corneal aspheric ablation profile. Previous studies have reported a significant postoperative increase in corneal epithelial thickness, which accounts for a slight regression of the net refractive power after several types of corneal refractive surgery [10–13]. However, few studies have investigated the contribution of epithelial hyperplasia in the correction of astigmatic corrections [14]. Furthermore, the presence of ocular residual astigmatism (ORA), defined as the vector difference between preoperative manifest refractive astigmatism and the astigmatism of the anterior cornea, can influence the efficacy of a variety of corneal refractive procedures for correcting myopic astigmatism when refractive correction is confined to the anterior cornea [15–17]. However, studies on the influence of the ORA on astigmatic correction using transPRK are limited [5]. We also measured corneal density (CD), which is positively correlated with intraocular straylight [18, 19], to explore its possible effect on the efficacy of transPRK [12].

Here, we evaluated the effects of CD, fixation stability, epithelial hyperplasia, and ORA on the efficacy of astigmatism correction using transPRK.

## Methods

This retrospective study included patients who underwent transPRK for the correction of myopia and myopic astigmatism between January 2023 and September 2023 at the Ophthalmology Department of the Eye and ENT Hospital, Shanghai, China. The inclusion criteria were as follows: age between 18 and 45 years; stable preoperative refraction (an annual increase of myopia less than 0.5 D for ≥ 2 years); manifest spherical equivalence of less than − 8.00 D; astigmatism greater than − 0.25 D and less than − 6.00 D; preoperative central corneal thickness greater than 470 µm, a calculated postoperative corneal thickness after ablation greater than 380 µm, and willingness to follow up regularly as required. Patients with any pathological ocular conditions, such as uveitis, cataract, glaucoma, history of any relevant systemic diseases, or history of prior ocular surgery, were excluded. Patients were divided into two groups according to refractive astigmatism [high refractive astigmatism (RA) group: ≥ 2.0 D, low RA group: < 2.0 D]. This study followed the tenets of the Declaration of Helsinki and was approved by the Ethics Committee of the EENT Hospital of Fudan University (reference number: 2023200). Written informed consent was obtained from all subjects.

### Surgical procedure

All treatment plans followed the Custom Ablation Manager protocol, and ablations were performed using an AMARIS 1050S excimer laser (SCHWIND Eye-Tech-Solutions, Kleinostheim, Germany) in the aberration-free mode (ablations were optimized to induce no change in wavefront aberration other than the sphere and cylinder components, leaving all existing HOAs unchanged). Proper alignment of the eye under the laser was achieved and centered on the corneal vertex using the pupillary offset determined by videokeratoscopy (Keratron Scout topographer, Optikon 2000, Italy) under photopic conditions (1500 lx) similar to those under the operating microscope. Automatic static cyclotorsion compensation and dynamic cyclotorsion control were used during surgery. Briefly, an eye registration module is included in the eye tracker subsystem, in which the diagnostic image from videokeratoscopy is used as a reference and is compared to an eye tracker image obtained before starting the ablation to determine the static cyclotorsion component. For dynamic cyclotorsion control, the first eye tracker image obtained before starting ablation was used as a reference and compared with any further eye tracker image to determine the dynamic cyclotorsion component during ablation. Corneal epithelial removal targeted the central 2 mm average epithelial thickness obtained from the anterior segment optical coherence tomography (AS-OCT, Optovue, Fremont, CA, USA) 6 mm epithelial map and 10 µm thicker than the central value at 8 mm peripherally [20], followed by stromal ablation in a single continuous profile. The attempted correction for spherical errors was based on the manifest refraction with a target of 0 to 0.50 D according to the age of the patient, and the attempted correction for cylindrical error was zero for all patients. Nomogram adjustment was implemented for the correction of cylindric error as follows: no adjustment for astigmatism less than 2.0 D; under-correction by 0.25 D for astigmatism between 2.0 and 3.0 D; and under-correction by 0.50 D for astigmatism greater than 3.0 D. The optical zone ranged between 6.3 and 7.0 mm based on the scotopic pupil size. After laser application, Mitomycin C 0.02% (0.2 mg/mL) was used for 10–45 s based on the depth of ablation. The eye was irrigated with 20 mL of balanced salt solution and a bandage contact lens was placed on the corneal surface for 7 days.

The postoperative treatment regimen included topical levofloxacin (Santen, Japan) four times daily until complete re-epithelialization, topical fluorometholone 0.1% (Santen, Japan) with a tapering regimen of 6, 5, 4, 3, 2, 1 times daily for 2 weeks, and sodium hyaluronate (Santen, Japan) four times daily for 3 months.

### Measurements

Patients were examined preoperatively and 1, 3, and 6 months postoperatively. The baseline and 6-month postoperative data were analyzed. Manifest and cycloplegic refraction tests were performed, and uncorrected distance visual acuity (UDVA) and corrected distance visual acuity (CDVA) in the logarithm of the minimum angle of resolution (logMAR) were recorded during all follow-up visits. Topometric measurements were performed by an experienced examiner using a Pentacam analyzer (Oculus GmbH, Wetzlar, Germany). The average of three measurements was used for each result.

### Vector analysis for astigmatism

ORA was defined as the vector difference between the preoperative RA and the anterior corneal astigmatism (AKA). Preoperative manifest RA was converted to the corneal plane using a vertex of 12 mm. The following indices were used: postoperative astigmatism determined using the manifest refraction (or difference vector; DV), target induced astigmatism vector (TIA), surgically induced astigmatism (SIA), correction index (CI), and angle of error (AOE). The CI is the ratio of SIA to TIA. A CI > 1 indicates over-correction, whereas a CI < 1 indicates under-correction. AOE is the angle between the SIA and TIA axes. An AOE with a positive value refers to a counterclockwise (cc/Wise) rotational error, whereas an AOE with a negative value refers to a clockwise (c/Wise) rotational error [21].

### Measurements of the lateral movement of the pupil center (PC) during ablation

**Fig. 1:**
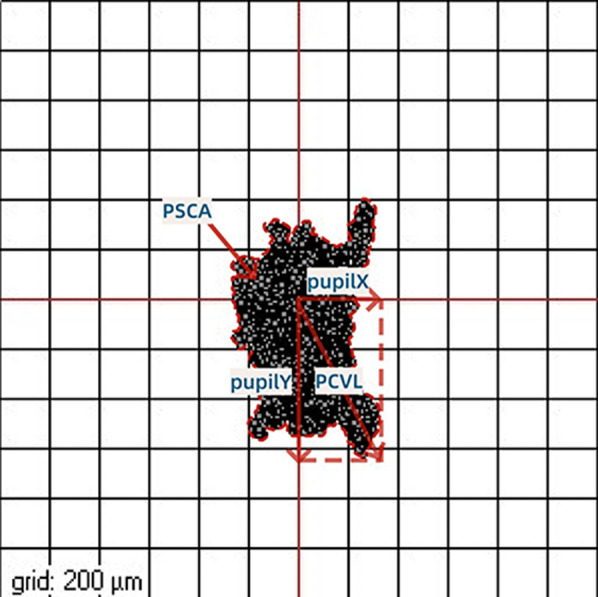
Measurements of the lateral movement of the pupil center during ablation. PupilX, maximum horizontal shift of pupil center; PupilY, maximum vertical shift of pupil; PCVL, vector length of pupil center shift; PCSA, the area covering the shift of pupil center

A pupil registration module is included in the eye tracker subsystem, in which the first pupil image obtained before starting the ablation is used as a reference, and its location is used for further eye-tracker images to determine the PC shift compensation [7]. The PC shift is represented in Cartesian coordinates (Pupil X and Pupil Y, Fig. 1), where Pupil X indicates the maximum horizontal shift of the PC and Pupil Y indicates the maximum vertical shift of the PC. The vector length of the PC shift (PCVL) was calculated as $\sqrt{{PupilX}^{2}+{PupilY}^{2}}$. The PC shift area was traced freehand, and the area surrounded by the outline was defined as the PC shift area (PCSA) and assessed using ImageJ software (Fiji, http://rsb.info.nih.gov/) [22].

### Measurements of corneal epithelial thickness and corneal densitometry

Epithelial thickness (ET) mapping was acquired by RTVue optical coherence tomography (Optovue, Fremont, CA, USA), and the postoperative change in the mean ET of 6 mm (ΔET = postop – preop ET of 6 mm) was used for analysis. The CD was measured using a Pentacam, and the output was expressed in grayscale units (GSU), which defined a minimum light scatter of 0 (maximum transparency) and a maximum light scatter of 100 (minimum transparency). The 12-mm diameter corneal area was subdivided into four concentric radial sectors: a central area of 2 mm centered on the apex, the first annulus extending from 2 to 6 mm, the second annulus extending from 6 to 10 mm, and the final annulus extending from 10 to 12 mm. CD was evaluated based on corneal depth in the anterior, middle, and posterior layers. The anterior layer corresponds to the anterior 120 μm, the posterior layer corresponds to the most posterior 60 μm, and the middle layer is between them. The central 2 mm of the anterior layer (CD_0-2A_), middle layer (CD_0-2 M_), posterior layer (CD_0-2P_), and 2–6 mm annuli of the anterior (CD_2-6A_), middle (CD_2-6M_), and posterior layers (CD_2-6P_) were used for the analysis.

### Statistical analyses

Statistical analyses were performed using the SPSS software (version 13.0; SPSS Inc., Chicago, IL, USA). The normality of the data was assessed using the Kolmogorov–Smirnov test. Differences between the pre- and postoperative outcomes were analyzed using a paired *t*-test for normally distributed data and the Wilcoxon signed-rank test for non-normally distributed parameters. Pearson’s correlation was used for normally distributed data and Spearman’s correlation for non-normally distributed data. Multivariate linear regression analyses were conducted for indices related to CI and AOE. Statistical significance was set at *P* < 0.05.

## Results

**Fig. 2:**
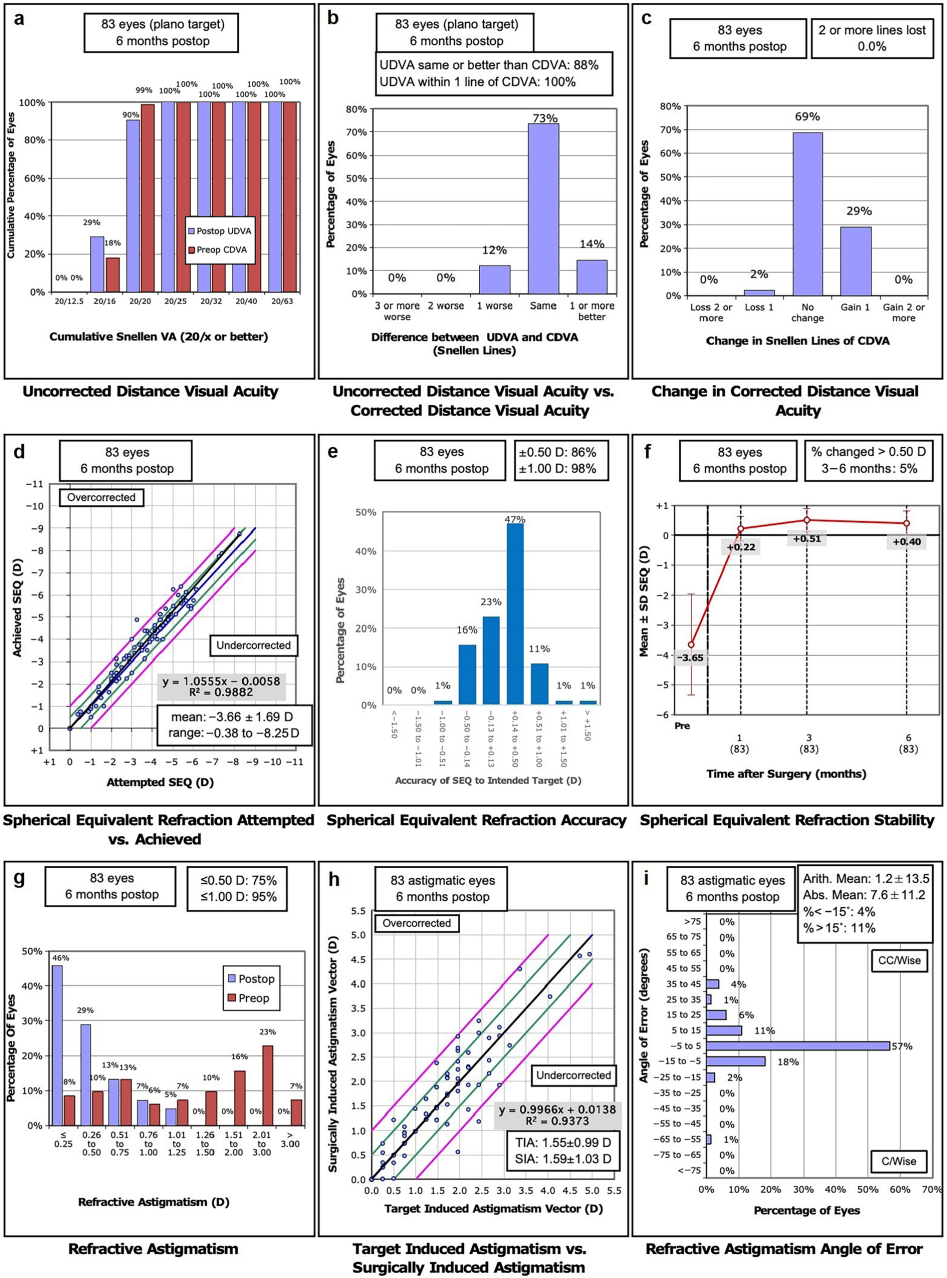
Refractive outcomes of transPRK at 6 months postoperatively. **a** Uncorrected distance visual acuity (UDVA); **b** UDVA vs. corrected distance visual acuity (CDVA); **c** Changes in CDVA; **d** Attempted vs. achieved spherical equivalent refraction (SEQ); **e** Accuracy of SEQ; **f** Stability of SEQ; **g** Refractive astigmatism (RA); **h** Target induced astigmatism vs. surgically induced astigmatism; **i** Angle of error. postop, postoperative

The right eyes of 45 males (54.2%) and 38 females (45.8%) were included in this study (high RA group, n = 31; low RA group, n = 52). The mean age was 27.40 ± 5.53 years (range: 18–44 years). The pre- and postoperative refractive data are listed in Table 1. The arithmetic means of pre- and post-operative RA were − 1.65 ± 1.11 D (− 5.5 to − 0.25 D) and − 0.43 ± 0.40 D (− 1.50 to 0.0 D), respectively (*P* < 0.001). The vector means were − 1.35 D × 179.0° and 0.08 D × 38.1° for the pre- and postoperative RA, respectively. The vector mean for the SIA was 1.34 × 177.3°. No clinical haze scale ≥ 0.5 was observed at 6 months postoperatively. The rate of successful registration of static cyclotorsion was 89.2% (74/83). The mean static cyclotorsion from upright to supine was − 0.12 ± 2.60° (range: − 6.3° to 6.8°). A total of 79.7% (59/74) of static cyclotorsion measurements were within 4° and 90.5% (67/74) of static cyclotorsion measurements were within 5°. The arithmetic mean of the ORA was 0.78 ± 0.35 D (0.01 to 1.77 D). The refractive results at 6 months postoperatively shown in Fig. 2 were based on the standard for reporting the astigmatism outcomes of refractive surgery [23].

**Table 1:** Preoperative and postoperative ocular characteristics

Parameter	Preoperative	6 months postoperative	*P* value
Sphere (D)	− 2.83 ± 1.80 (− 7.25, 0.50)	0.44 ± 0.44 (− 0.25, 2.00)	< 0.001
RA (D)	− 1.65 ± 1.11 (− 5.50, − 0.25)	− 0.43 ± 0.40 (− 1.50, 0.00)	< 0.001
AKA (D)	2.18 ± 1.08 (0.06, 5.32)	0.88 ± 0.56 (0.45, 2.11)	< 0.001
TP (μm)	530.8 ± 31.5 (471, 599)	452.8 ± 43.6 (390, 549)	< 0.001
UDVA (logMAR)	0.87 ± 0.25 (0.1, 1.0)	− 0.02 ± 0.06 (− 0.1, 0.1)	< 0.001
CDVA (logMAR)	− 0.02 ± 0.04 (− 0.1, 0.1)	− 0.04 ± 0.05 (− 0.2, 0.1)	< 0.001
Corneal aberrations			
Total HOA (µm)	0.32 ± 0.14 (0.11, 1.01)	0.51 ± 0.19 (0.18, 1.01)	< 0.001
Vertical coma (µm)	0.03 ± 0.16 (− 0.37, 0.35)	0.01 ± 0.23 (− 0.52, 0.59)	0.584
Horizontal coma (µm)	0.01 ± 0.20 (− 0.58, 0.61)	0.10 ± 0.21 (− 0.54, 0.52)	< 0.001
Spherical aberration (µm)	0.04 ± 0.10 (− 0.26, 0.24)	0.15 ± 0.24 (− 0.49, 0.75)	< 0.001
Mean epithelial thickness of 6 mm (µm)	53.19 ± 2.58 (48.50, 61.00)	56.05 ± 4.10 (44.00, 66.00)	< 0.001
Corneal density			
CD_0-2A_	21.48 ± 1.95 (16.10, 26.30)	22.35 ± 1.67 (18.80, 26.70)	< 0.001
CD_0-2M_	13.30 ± 0.77 (10.80, 15.10)	15.30 ± 0.93 (12.50, 17.30)	0.001
CD_0-2P_	10.23 ± 2.22 (6.10, 13.80)	10.32 ± 0.98 (8.10, 12.90)	0.527
CD_2-6A_	19.40 ± 1.84 (14.60, 23.50)	20.03 ± 1.53 (16.80, 23.10)	< 0.001
CD_2-6M_	12.11 ± 0.63 (9.90, 13.70)	13.42 ± 0.81 (11.60, 15.30)	< 0.001
CD_2-6P_	9.54 ± 1.91 (5.90, 12.70)	9.89 ± 0.81 (8.10, 12.20)	0.199

*RA* = refractive astigmatism; *AKA* = anterior corneal astigmatism; *TP* = thinnest pachymetry; *UDVA* = uncorrected distance visual acuity; *CDVA* = corrected distance visual acuity; *HOA* = high-order aberration; *CD*_*0-2A*_ = corneal density of central 2 mm of the anterior layer; *CD*_*0-2M*_ = corneal density of central 2 mm of the middle layer; *CD*_*0-2P*_ = corneal density of central 2 mm of the posterior layer; *CD*_*2-6A*_ = corneal density of 2–6 mm of the anterior layer; *CD*_*2-6M*_ = corneal density of 2–6 mm of the middle layer; *CD*_*2-6P*_ = corneal density of 2–6 mm of the posterior layer

The pre-and postoperative corneal aberrations at 6 mm are shown in Table 1. The total HOA (*P* < 0.001), horizontal coma (*P* < 0.001), and spherical aberration (*P* < 0.001) significantly increased postoperatively.

### Corneal epithelial thickness and corneal densitometry

The pre- and postoperative ET values at 6 mm and the CDs are shown in Table 1. The ET was significantly thickened postoperatively (Wilcoxon signed-rank test: *P* < 0.001). Corneal densitometry values increased significantly postoperatively for CD_0-2A_ (*P* < 0.001), CD_0-2M_ (*P* = 0.001), CD_2-6A_ (*P* < 0.001), and CD_2-6M_ (*P* < 0.001).

### Lateral movement of PC during ablation

The mean maximum horizontal (PupilX) and vertical (PupilY) shifts of the PC were 0.56 ± 0.24 mm (0.20 to 1.18 mm) and 0.72 ± 0.29 mm (0.20 to 1.18 mm), respectively. A positive correlation was observed between PupilX and PupilY levels (Spearman correlation coefficient: r = 0.319, *P* = 0.003). In total, 50.6% of PupilX and 25.3% of PupilY were within 0.5 mm. Furthermore, 9.6% of Pupil X and 19.3% of Pupil Y were greater than 1 mm in size. The mean vector length of PCVL was 0.93 ± 0.31 mm (0.32 to 1.62 mm) and the mean area of PC shift (PCSA) was 0.38 ± 0.22 mm^2^ (0.08 to 1.22 mm^2^). A positive correlation was found between the PCVL and PCSA (Spearman’s correlation coefficient: r = 0.781, *P* < 0.001).

### Comparisons between high and low RA groups

The results of the comparisons between the high and low RA groups are shown in Table 2. The residual RA was greater in the high RA group (− 0.66 ± 0.44 D vs. − 0.29 ± 0.29 D, *P* < 0.001) and the postoperative UDVA (*P* = 0.006) and CDVA (*P* < 0.001) were better in the low RA group. No significant differences were found in CI or AOE between the two groups (*P* > 0.05). ΔET was greater in the low RA group (0.68 ± 3.47 µm vs. 3.85 ± 3.08 µm, *P* < 0.001), and ΔCD_2-6A_ was higher in the high RA group (1.14 ± 1.78 vs. 0.27 ± 1.53, *P* = 0.021).

**Table 2:** Comparisons between eyes with refractive astigmatism higher and lower than 2.0 D

Parameter	High RA group (n = 31)	Low RA group (n = 52)	*P* value
Sphere (D)	− 2.05 ± 1.95 (− 7.25, 0.50)	− 3.29 ± 1.54 (− 6.50, 0.00)	0.004
RA (D)	− 2.79 ± 0.88 (− 5.50, − 2.00)	− 0.97 ± 0.51 (− 1.75, 0.00)	< 0.001
SCC (°)	0.53 ± 2.56 (− 5.08, 6.80)	− 0.51 ± 2.58 (− 6.30, 5.30)	0.677
PCVL (mm)	0.92 ± 0.33 (0.33, 1.62)	0.94 ± 0.30 (0.32, 1.50)	0.658
Postoperative RA	− 0.66 ± 0.44 (− 1.50, 0.00)	− 0.29 ± 0.29 (− 1.00, 0.00)	< 0.001
CI	1.01 ± 0.26 (0.29, 1.50)	1.06 ± 0.45 (0.03, 2.95)	0.567
AOE	− 0.88 ± 5.35 (− 9.92, 13.50)	2.42 ± 16.51 (− 56.19, 44.50)	0.188
Postoperative UDVA (logMAR)	0.00 ± 0.05 (− 0.1, 0.1)	− 0.03 ± 0.06 (− 0.1, 0.1)	0.006
Postoperative CDVA (logMAR)	− 0.02 ± 0.05 (− 0.1, 0.1)	− 0.06 ± 0.05 (− 0.2, 0.0)	< 0.001
ΔTotal HOA (µm)	0.21 ± 0.20 (− 0.25, 0.78)	0.16 ± 0.21 (− 0.22, 0.60)	0.363
ΔVertical coma (µm)	0.07 ± 0.28 (− 0.59, 0.89)	− 0.04 ± 0.18 (− 0.56, 0.41)	0.074
ΔHorizontal coma (µm)	0.12 ± 0.22 (− 0.26, 0.47)	0.07 ± 0.20 (− 0.34, 0.79)	0.399
ΔSpherical aberration (µm)	0.02 ± 0.26 (− 0.49, 0.83)	0.15 ± 0.19 (− 0.27, 0.47)	0.028
Δ ET of 6 mm (µm)	0.68 ± 3.47 (− 7.00, 8.00)	3.85 ± 3.08 (− 4.00, 12.00)	< 0.001
ΔCD_0-2A_	1.22 ± 2.11 (− 3.00, 6.00)	0.66 ± 1.51 (− 2.20, 4.50)	0.162
ΔCD_0-2M_	2.06 ± 1.21 (0.10, 5.40)	1.97 ± 0.83 (− 0.60, 3.90)	0.670
ΔCD_0-2P_	0.59 ± 2.22 (− 3.70, 5.10)	− 0.21 ± 2.61 (− 3.90, 6.40)	0.087
ΔCD_2-6A_	1.14 ± 1.78 (− 1.90, 5.30)	0.27 ± 1.53 (− 2.50, 4.10)	0.021
ΔCD_2-6M_	1.32 ± 0.97 (− 0.50, 3.80)	1.31 ± 0.69 (− 0.20, 3.00)	0.935
ΔCD_2-6P_	0.70 ± 1.89 (− 3.20, 4.60)	0.13 ± 2.33 (− 3.10, 5.70)	0.120

*RA* = refractive astigmatism; *SCC* = static cyclotorsion compensation; *PCVL* = vector length of pupil center shift; *CI* = correction index; *AOE* = angle of error; *UDVA* = uncorrected distance visual acuity; *CDVA* = corrected distance visual acuity; *HOA* = high-order aberration; *ET* = epithelial thickness; *CD*_*0-2A*_ = corneal density of central 2 mm of the anterior layer; *CD*_*0-2M*_ = corneal density of central 2 mm of the middle layer; *CD*_*0-2P*_ = corneal density of central 2 mm of the posterior layer; *CD*_*2-6A*_ = corneal density of 2–6 mm of the anterior layer; *CD*_*2-6M*_ = corneal density of 2–6 mm of the middle layer; *CD*_*2-6P*_ = corneal density of 2–6 mm of the posterior layer; *Δ* = postoperative − preoperative

### Factors associated with CI and AOE

The results of multiple linear regression analyses evaluating the association between individual parameters and CI and AOE in the low RA group are shown in Table 3. The results revealed that ΔET (*P* = 0.041) and preoperative CD_0-2A_ (*P* = 0.011) were associated with CI, whereas PCVL (*P* = 0.005) and ΔET (*P* = 0.036) were associated with AOE.

**Table 3:** Results of multiple linear regression analysis for eyes with refractive astigmatism less than 2.0 D

Parameter	CI		AOE	
	Standardized β	*P* value	Standardized β	*P* value
Age	− 0.136	0.459	− 0.232	0.193
Sex	− 0.160	0.344	0.163	0.317
Sphere	− 0.012	0.935	− 0.095	0.503
Cylinder	0.109	0.426	0.226	0.093
Astigmatic type	− 0.174	0.270	0.063	0.676
ORA	− 0.155	0.292	0.202	0.156
PCVL	0.264	0.067	− 0.404	0.005
Static cyclotorsion	0.184	0.234	− 0.225	0.135
ΔET	0.295	0.041	− 0.293	0.036
Preop CD_0-2A_	− 0.482	0.011	0.284	0.112
Preop CD_0-2M_	0.096	0.569	− 0.031	0.846

*CI* = correction index; *AOE* = angle of error; *ORA* = ocular residual astigmatism; *PCVL* = vector length of pupil center shift; *ΔET* = postop epithelial thickness – preop epithelial thickness of 6 mm; *CD*_*0-2A*_ = corneal density of central 2 mm of the anterior layer; *CD*_*0-2M*_ = corneal density of central 2 mm of the middle layer

The results of the multiple linear regression analyses evaluating the association between individual parameters and CI and AOE in the high RA group are shown in Table 4. The results showed that ΔET (*P* = 0.041) was associated with CI, and static cyclotorsion compensation (*P* = 0.040) was associated with AOE.

**Table 4:** Results of multiple linear regression analysis for eyes with refractive astigmatism higher than 2.0 D

Parameter	CI		AOE	
	Standardized β	*P* value	Standardized β	*P* value
Age	0.512	0.058	0.117	0.648
Sex	− 0.299	0.212	− 0.255	0.281
Sphere	− 0.269	0.225	0.075	0.730
Cylinder	− 0.117	0.622	0.174	0.460
Astigmatic type	− 0.196	0.399	0.168	0.466
ORA	0.452	0.091	− 0.428	0.106
PCVL	− 0.003	0.987	0.042	0.835
Static cyclotorsion	0.022	0.925	− 0.493	0.040
ΔET	0.519	0.038	0.117	0.619
Preop CD_0-2A_	0.209	0.399	− 0.051	0.835
Preop CD_0-2M_	− 0.240	0.369	0.050	0.850

*CI* = correction index; *AOE* = angle of error; *ORA* = ocular residual astigmatism; *PCVL* = vector length of pupil center shift; *ΔET* = postop epithelial thickness – preop epithelial thickness of 6 mm; *CD*_*0-2A*_ = corneal density of central 2 mm of the anterior layer; *CD*_*0-2M*_  = corneal density of central 2 mm of the middle layer

## Discussion

The current study evaluated the refractive and visual outcomes of the transPRK and found that it was effective in terms of UDVA, CDVA, and spherical and astigmatic corrections, with a limited increase in HOAs. No significant differences were found in the CI or AOE between the high and low RA groups. Multiple linear regression analyses showed that in the low RA group, preoperative anterior CD and postoperative change in ET were associated with CI, whereas intraoperative shift of the PC and postoperative change in ET were associated with AOE. In the high RA group, postoperative changes in ET were associated with CI, whereas static cyclotorsion was associated with AOE. No significant associations were found between ORA and CI or AOE. To the best of our knowledge, this study is the first to report the contributions of pupil shift and CD to correcting astigmatism using transPRK. Moreover, the ET results confirmed the refractive contribution of epithelial mapping to the astigmatic component.

Natural eye movement and improper fixation cannot be avoided during refractive surgery. There are several types of eye movements, including saccades, drifts, microsaccades, tremors, and head movements. Active eye-tracking systems have been introduced to compensate for these movements and reduce the effects of misaligned laser shots. Bueeler et al. investigated the lateral alignment accuracy required in wavefront-guided refractive surgery and found that to achieve the diffraction limit in 95% of normal eyes with a 7.0-mm pupil, a lateral alignment accuracy of 70 μm or better was required. An accuracy of 200 μm was sufficient to reach the same goal with a 3.0-mm pupil [24]. The accuracy of eye tracking depends on several factors, such as the geometric parallax between the PC, corneal apex, and eye-tracker position; the contrast between the pupil and iris provided by illumination; and the latency between the measurements of eye movements and adjustment of the laser [25, 26]. This study revealed that poor fixation during ablation affected the efficacy of transPRK astigmatism correction, particularly in eyes with low astigmatism. The correction of low astigmatism is more susceptible to various perioperative influencing factors [27], and this could also be corroborated by the larger standard deviations in both CI and AOE in the low RA group. A significant association was observed between static cyclotorsion and AOE in patients with high astigmatism. This demonstrates that accurate static cyclotorsion compensation is essential for correcting high astigmatism [28].

Corneal densitometry was quantitatively assessed, and a significant correlation was found between preoperative central CD and CI in the low RA group. This result highlights the influence of CD on forward scattering in the cornea and, consequently, on the efficacy of laser ablation. However, the underlying mechanisms are not well understood. Previous studies have revealed a positive correlation between CD and intraocular straylight [18, 19]. In humans, the central stroma is composed of about 200 to 250 stacked lamellae that lie essentially parallel to the corneal surface. The specific arrangement of collagen in the human corneal stroma produces destructive interference of light scattered in all directions other than the forward direction, leading to tissue transparency [29]. Variations in collagen arrangement and the degree of hydration may be related to individual differences in corneal clarity, resulting in varied intraocular scattering. Stromal refractive index and hydration affect postoperative outcomes, indicating that changes in the structure of the cornea may lead to changes in the absorption coefficient or ablation threshold [30, 31]. Therefore, customization using individual refractive indices and hydration measurements should be considered when planning surgery and modifying the ablation algorithm. Further studies are required to elucidate the underlying mechanisms.

In addition, a significant increase in the ET of the central 6 mm region was observed 6 months postoperatively in this study. The interaction between the epithelia and keratocytes during healing can contribute to changes in ET, as reported in previous studies [12, 32]. The correlations between ΔET and the efficacy of astigmatic correction for both high and low RA groups demonstrate the contribution of epithelial remodeling on the correction of astigmatism in transPRK [33]. It is generally believed that the corneal epithelium can alter its thickness profile to reduce surface irregularities, which have been demonstrated in eyes undergoing refractive surgery or wearing orthokeratology [34–36]. Thickening of the epithelium has been observed in the eyes after transPRK, which positively correlated with the surgically induced change in the Q-value [33]. Moreover, heterogeneous epithelial hyperplasia with greater thickening along the flat axis was observed in eyes with high with-the-rule astigmatism after small incision lenticule extraction (SMILE) [14]. Further studies will be conducted to compare the patterns of epithelial proliferation among different types of surgical procedures.

Significant improvement was observed in sphere, cylinder, UDVA, and CDVA at 6 months postoperatively in this cohort. Horizontal coma and spherical aberrations increased slightly, whereas no significant changes were observed in vertical coma. These results confirm the accuracy of the centration pattern and the reliable performance of aberration-free profiles using transPRK. A slight overcorrection was observed in the cylinder even in the case of nomogram adjustment [5, 37]. A longer follow-up period is required to further optimize nomogram adjustment. However, no correlation was found between the ORA and CI or AOE in this study. The report by de Ortueta et al. also explored the influence of ORA on the efficiency of correcting astigmatism by transPRK and found no significant difference between the low and high ORA subgroups, except for a change in the Snellen lines of the CDVA [5]. Differences in the findings between transPRK and other procedures could arise from differences in ablation profiles.

This study has several limitations. First, its retrospective nature imposed natural limitations on the study cohort. Second, only X/Y lateral pupil shifts were measured to evaluate fixation stability. Third, the variable optical zone for stromal ablation (6.3–7.0 mm) may affect the correction efficacy. Finally, the 6-month follow-up period was relatively short, and the long-term effects of TPRK on astigmatism should be explored through further follow-up.

## Conclusions

TransPRK was effective for UDVA, CDVA, and spherical and astigmatic corrections, with a limited increase in HOAs. The postoperative change in the epithelial thickness was related to the efficacy of transPRK in both low and high RA groups, whereas the PC shift and anterior CD were associated with the efficacy of transPRK in the low RA group.

## Data Availability

The datasets used and analyzed during the current study are available from the corresponding author on reasonable request.

## Abbreviations

AKA: Anterior corneal astigmatism
AOE: Angle of error
CD: Corneal density
CDVA: Corrected distance visual acuity
CI: Correction index
ET: Epithelial thickness
ΔET: The postoperative change in the epithelial thickness of 6 mm
HOAs: Higher-order aberrations
ORA: Ocular residual astigmatism
PC: Pupil center
PCSA: The area of pupil center shift
PCVL: The vector length of the PC shift
Pupil X: The maximum horizontal shift of the PC
Pupil Y: The maximum vertical shift of the PC
RA: Refractive astigmatism
SIA: Surgically induced astigmatism
TIA: Target-induced astigmatism
transPRK: Transepithelial photorefractive keratectomy
UDVA: Uncorrected distance visual acuity
